# Assessment of Phenotypic Tools for Detection of OXA-48, KPC, and NDM in *Klebsiella pneumoniae* in Oman

**DOI:** 10.3390/diagnostics15080949

**Published:** 2025-04-08

**Authors:** Arwa AL Rujaibi, Zaaima AL Jabri, Amina Al Jardani, Azza AL Rashdi, Azza AL Mamari, Sara AL Sumri, Hiba Sami, Zakariya Al Muharrmi, Meher Rizvi

**Affiliations:** 1 Department of Microbiology and Immunology, College of Medicine and Health Sciences, Sultan Qaboos University, Muscat 123, Oman; s52151@student.squ.edu.om (A.A.R.); zaeema@squ.edu.om (Z.A.J.); s33978@student.squ.edu.om (A.A.M.); 2 Central Public Health Laboratories, Center for Disease Control and Prevention, Ministry of Health, Muscat 132, Oman; aksaljardani@gmail.com (A.A.J.); a.rashdi24@gmail.com (A.A.R.); arwaslab@gmail.com (S.A.S.); 3 Department of Microbiology, Jawaharlal Nehru Medical College, Aligarh Muslim University, Aligarh 202001, India; hibasamizafar@gmail.com; 4 Department of Microbiology and Immunology, Sultan Qaboos University and University Medical City, Muscat 123, Oman; muharrmi@squ.edu.om

**Keywords:** OXA-48 disk test, ICT, D73C, Gene Xpert, WGS, *Klebsiella pneumoniae*, OXA-48, KPC, NDM

## Abstract

**Background**: The alarming increase in carbapenemase-producing Enterobacterales is a matter of grave public health concern. The most ubiquitous carbapenemases, *Klebsiella pneumoniae* carbapenemase (KPC)-, New Delhi metallo-β-lactamase (NDM)-, and oxacillinase (OXA-48)-like enzymes, belong to the Ambler molecular classes A, B, and D, respectively. KPC- and OXA-48-like enzymes have a serine-based hydrolytic mechanism, while NDMs are metallo-β-lactamases that contain zinc in the active site. For the judicious use of reserve drugs and promoting antimicrobial stewardship, timely detection of carbapenemases is essential. While molecular tools are the gold standard for the detection of these enzymes, many laboratories have limited access to them. This study focused on evaluating in-house tools and commercial phenotypic tests for the detection of OXA-48-, KPC-, and NDM-like enzymes in *K. pneumoniae*, the predominant extremely drug-resistant pathogen in Oman. **Methods**: In total, 80 GeneXpert/PCR-confirmed (40 OXA-48 and 20 KPC and NDM each) and 37 whole-genome-sequenced (25 OXA-232 and 6 KPC-2, plus NDM-1 and NDM-5) *K. pneumoniae* were subjected to screening by temocillin (30 μg disk) (MAST Diagnostica, Germany) and D71C (MASTDISCS^®^). Isolates resistant to temocillin (<11 mm) and D71C were subjected to four tests: an in-house tool (OXA-48 disk test) and three commercial phenotypic tests: (i) the MASTDISCS^®^ Combi (D72C) (MAST Group Ltd., Bootle, UK); (ii) the MASTDISCS^®^ Combi (D73C) (MAST Group Ltd., UK); and (iii) an immunochromatographic assay (ICT), which is the KPC/IMP/NDM/VIM/OXA-48 Combo test kit (Medomics, China), for the detection of OXA-48-, KPC-, and NDM-like carbapenemases. **Results**: Temocillin exhibited good sensitivity and specificity (100% and 97.50%) compared to D71C (70% and 100%). Among the confirmatory tests, the in-house OXA-48 disk test had 92.50% sensitivity and 100% specificity, while the commercial MAST DISC tests D72C, D73C, and ICT had 97.50%, 95.00%, and 100% sensitivity and 100%, 91.67%, and 95% specificity, respectively. **Conclusions**: The temocillin disk test is a good screening tool. With high sensitivity and specificity, ease of performance, short turnaround time, and low cost, we recommend the ICT format for routine diagnostic use. In resource-constrained centers, the OXA-48 disk test is an excellent alternative with high sensitivity and specificity.

## 1. Introduction

The emergence of carbapenemase-producing Enterobacterales (CPEs) is undoubtedly one of the most significant health challenges of the century [1]. Carbapenemases are recognized as the most versatile β-lactamases, with a hydrolytic spectrum that surpasses that of extended-spectrum and AmpC β-lactamases. According to Ambler’s classification, they are categorized into three classes: A, B, and D. Within Ambler class A, plasmid-encoded carbapenemases include KPC (KPC-1 to KPC-13), IMI (IMI-1 to IMI-3), and GES (Guiana extended spectrum) (GES-1 to GES-20), whereas chromosomally encoded carbapenemases include Not Metalloenzyme Carbapenemase A (NMC-A) and Serratia marcescens enzyme (SME). Class B carbapenemases are β-lactamases capable of hydrolyzing carbapenems but can be inhibited by ethylenediaminetetraacetic acid (EDTA), a chelator of Zn^2+^ and other divalent cations. Prominent metallo-β-lactamase families in this class include New Delhi metallo-β-lactamase-1 (NDM-1), imipenem-resistant Pseudomonas (IMP)-type carbapenemases, Verona integron-encoded metallo-β-lactamase (VIM), German imipenemase (GIM), and Seoul imipenemase (SIM). NDM genes are primarily found in Klebsiella pneumoniae and Escherichia coli isolates but have also been detected in association with Acinetobacter baumannii and Pseudomonas aeruginosa. Class D carbapenemases, which are serine-β-lactamases, exhibit poor inhibition by EDTA or clavulanic acid and are classified as OXA enzyme types (oxacillin carbapenemases) with limited activity against carbapenems [2].

The incidence of these enzymes is on the rise in comparison to the total infections caused by Enterobacterales. Amongst all the members of this family, CPE predominates in Klebsiella species [3,4,5,6,7]. In the Middle East, carbapenem resistance among Enterobacterales isolates is 4.3% [8]. The rapid detection of carbapenemases and the initiation of appropriate antimicrobials improve patient outcomes, strengthen antimicrobial stewardship efforts, and allow the judicious use of the reserve antibiotics [9]. Studies indicate that, as with other carbapenemases, OXA-48-producing strains are associated with poor patient outcomes. However, OXA-48 detection is rather challenging, due to low-level hydrolytic activity toward carbapenems and no intrinsic activity against expanded-spectrum cephalosporins [7]. While molecular methods are considered the gold standard for the detection of CPE, their accessibility is limited, particularly in resource-constrained settings [10]. It is important to evaluate phenotypic tests for the detection of carbapenemases. Such tests enable their detection at scale, allow the optimization of treatment, and enhance surveillance and infection prevention and control efforts. The temocillin disk test has been described in many studies as a useful tool for the detection of OXA-48, in conjunction with ertapenem or piperacillin–tazobactam [11,12]. Dijk et al. recommended the temocillin test along with carbapenemase inhibition tests (PBA and DPA) as a confirmatory tool for class A, B, and D carbapenemases in Enterobacterales [13]. Tsakris et al. recommended the OXA-48 disk test for accurate phenotypic detection of OXA-48-producing Enterobacterales [14]. The Mast group with D71, D72, and D73 is a useful tool for the detection of extended-spectrum beta lactamases (ESBLs), AmpC beta lactamases, and CPE [15]. Several good lateral flow immunoassay (LFIA) tests are available in the market, like KINVO (Medomics Medical Technology, Nanjing, Jiangsu, China), the O.K.N.V.I RESIST-5 (RESIST-5, Coris BioConcept, Gembloux, Belgium), and the NG-Test^®^ DetecTooL CARBA 5 (CARBA-5, NG Biotech, Guipry-Messac, France). They are rapid, easy to use, and relatively cheap [16]. In this study, we assessed the temocillin disk test and D71C as screening tests and the OXA disk test, D72C, D73C, and an LFIA (Medomics) as confirmatory tests. This was carried out in an effort to identify sensitive, specific, and cost-effective tests for the detection of OXA-48, KPC, and NDM in *K. pneumoniae*, which may be of use in low-resource settings as well.

## 2. Materials and Methods

### 2.1. Sample Collection

A collaborative study was initiated between Sultan Qaboos University Hospital (SQUH) and the Central of Public Health Laboratories (CPHL), Oman, with the purpose of identifying a sensitive, specific, and cost-effective test for the detection of OXA-48-, KPC-, and NDM-like enzymes in *K. pneumoniae* which may be useful in low-resource settings as well. One thousand and fifty-five clinical isolates of *K. pneumoniae* obtained from SQUH and CPHL were screened for OXA-48-, KPC-, and NDM-like enzymes over a six-month period. Four hundred and thirteen consecutive, non-duplicate (i) meropenem and imipenem resistant isolates; (ii) isolates which demonstrated intermediate MICs to carbapenems; (iii) isolates with resistance to piperacillin–tazobactam; and (iv) isolates that were susceptible, intermediate, or resistant to third-generation cephalosporins [17] were subjected to genotypic confirmation by Xpert ^®^Carba-R, PCR, and WGS which are considered the gold standard methods for identifying CPE.

### 2.2. Genotypic Confirmation of Carbapenemases by Gene Xpert/PCR

The isolates were subjected to Gene Xpert ^®^Carba-R (Cepheid, Sunnyvale, CA, USA) in SQUH and to a standardized PCR protocol (Platinum multiplex PCR master mix, Thermofisher, Vilnius, Lithuania) in CPHL [18]. In brief, at CPHL, the molecular analysis involved testing for the presence of carbapenemase genes: *bla*KPC, *bla*NDM, *bla*OXA-48, *bla*IMP, and *bla*VIM-like enzymes. A pure isolate was used to extract the DNA, using QIAsymphony DSP DNA Mini Kit as per the manufacturer’s instructions. Multiplex PCR targeted *bla*OXA-48, *bla*NDM, *bla*KPC, *bla*IMP, and *bla*VIM [19]. The primers were obtained from Eurofins Genomics, Ebersberg, Germany, GmbH. Details are provided in Tables S1–S3 of the Supplementary File. Gel electrophoresis was used for the molecular characterization of the CPE genes.

### 2.3. Whole-Genome Sequencing (WGS)

Whole-genome sequencing (WGS) was performed at MicrobesNG using Illumina next-generation sequencing for representative OXA-48, KPC, and NDM in *K. pneumoniae* strains (https://microbesng.co.uk, accessed on 14 September 2022, Birmingham, UK). The DNA samples were prepared and sequenced according to the manufacturer’s protocol.

The extracted DNA was prepared following the manufacturer’s protocol as described on the company’s website as follows: ILLUMINA SEQUENCING (SGS and EGS) Genomic DNA libraries are prepared using the Nextera XT Library Prep Kit (Illumina, San Diego, CA, USA) following the manufacturer’s protocol with the following modifications: input DNA is increased 2-fold, and PCR elongation time is increased to 45 s. DNA quantification and library preparation are carried out on a Hamilton Microlab STAR automated liquid handling system (Hamilton Bonaduz AG, Bonaduz, Switzerland). Pooled libraries are quantified using the Kapa Biosystems Library Quantification Kit for Illumina. Libraries are sequenced using Illumina sequencers (HiSeq/NovaSeq) using a 250 bp paired-end protocol. Reads are adapter-trimmed using Trimmomatic 0.30 with a sliding window quality cutoff of Q15. Post-sequencing, the samples were assembled using the De Novo assembly by the SPAdes program [20]. The assembled contigs were annotated with Prokka [21] and uploaded to the website for further analysis. Multilocus sequence typing (MLST) v2.0.9 was determined using the MLST tool in the Center for Genomic Epidemiology (CGE) (https://www.genomicepidemiology.org/services/, accessed on September 2022) [22,23]. PlasmidFinder and ResFinder in the CGE website were used to identify plasmids and acquired antimicrobial resistance genes, respectively [24,25]. Additionally, the Comprehensive Antibiotic Resistance Database v3.2.8 (CARD) (https://card.mcmaster.ca/, accessed on December 2022) was used to detect putative antimicrobial resistance genes using the Resistance Gene Identifier (RGI) tool v6.0.3. This tool identifies the antibiotic resistome(s) as well as point mutations within the resistance-conferring genes [26]. Furthermore, the phylogeny tool in the CGE server was used to investigate the genetic relatedness between the different sequences by identifying single-nucleotide polymorphisms (SNPs).

From the above-mentioned pool, 40 OXA-48-like isolates, 20 each of NDM and KPC carbapenemase producers, and 37 whole-genome-sequenced isolates (25 OXA-232 and 6 KPC-2, plus NDM-1 and 6 NDM-5) were selected for evaluating the tests. OXA-232 harbored CTX-M-15, SHV-11, SHV-28, SHV-67, SHV-75, SHV106, and SHV-182, while the KPC-2+NDM-1 carried CTMX-65, SHV-12, SHV-12, SHV-182, and SHV-182 and NDM-5 carried CTX-M-65, TEM-1B, TEM-1C, SHV-11, SHV-12, SHV-67, and SHV-75.

CPE confirmed isolates were subjected to the following phenotypic tests for the detection of CPE.

### 2.4. Phenotypic Testing for Screening and Confirmation of CPE

**Figure 1:**
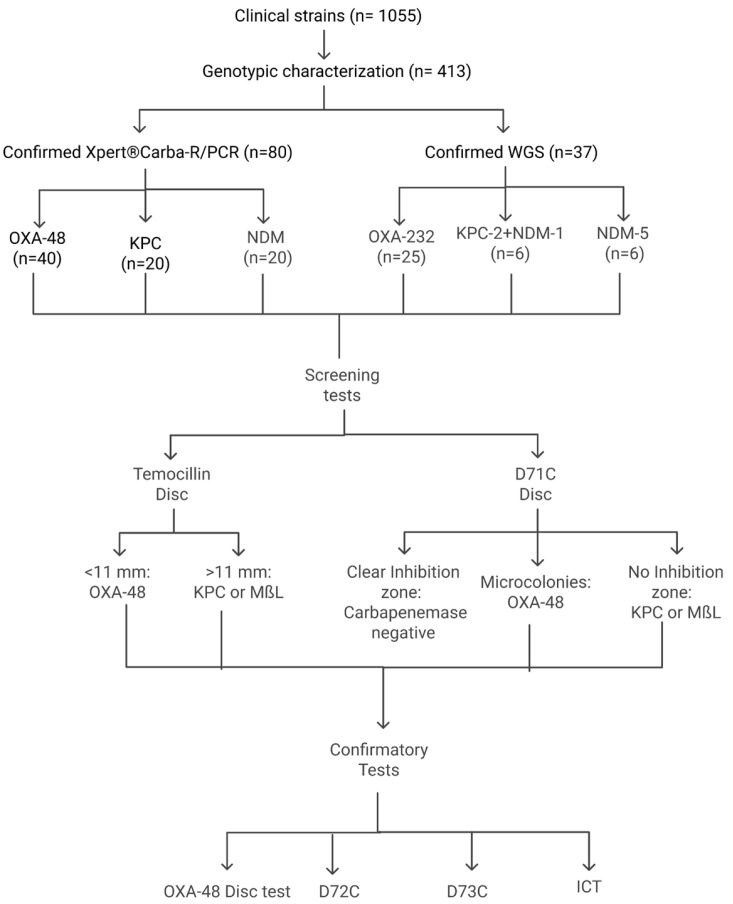
Algorithm for screening and confirmation of carbapenemases.

All isolates were first screened and then subjected to four confirmatory phenotypic tests for the detection of CPE. Figure 1 shows the algorithm followed. The details of each test are mentioned below. The cost of each test is provided in Table S4, Supplementary File.

### 2.5. Screening Test

#### Temocillin and D71C Disk Test

**Figure 2:**
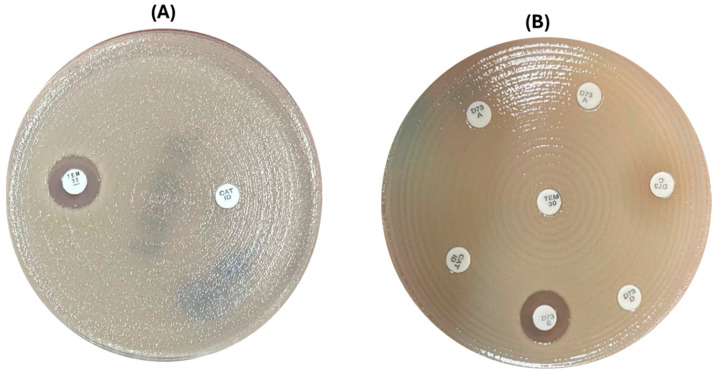
Phenotypic tests used for the detection of CPE. (**A**) The screening tests (*temocillin* and *D71C*), where the temocillin inhibition zone was >11 mm with no zone of inhibition around D71C (which was indicative of MBL or KPC). (**B**) Screening plus confirmatory tests for NDM detection: temocillin < 11 mm with no zone of inhibition around D71C, and the confirmatory D73C was positive for NDM.

A 30 μg temocillin disk (MAST Group Ltd., UK) and a faropenem disk from D71C (MAST Group Ltd., UK) were placed on a Mueller–Hinton plate inoculated with a fresh bacterial suspension (0.5 McFarland standard). Plates were incubated for 16 to 20 h under aerobic conditions at 35 °C. An inhibition zone diameter of 11 mm for temocillin was considered positive for OXA-48, KPC, or NDM [27,28]. The MAST^®^ CAT-ID (D71C) was interpreted according to the manufacturer’s recommendation as follows: a clearly defined zone of inhibition was indicative of no carbapenemase activity; no zone of inhibition was indicative of MBL or KPC; and single colonies in the zone of inhibition/double zone of inhibition were considered OXA-48-positive [29] (Figure 2).

### 2.6. Confirmatory Tests

#### 2.6.1. OXA-48 Disk Test

**Figure 3:**
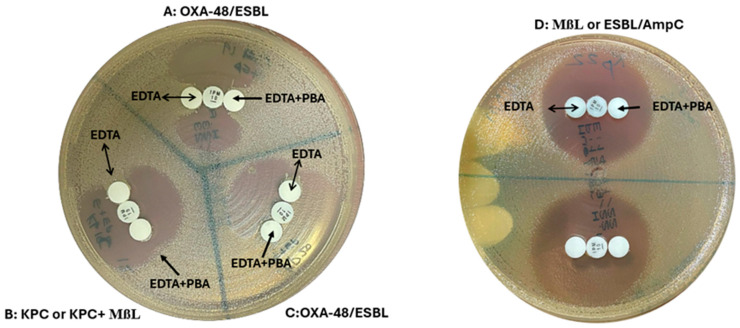
OXA-48 disk test. (**A**,**C**) Indentation towards both EDTA and EDTA/PBA disks indicated OXA-48 carbapenemase production. (**B**) Indentation towards the EDTA disk but not the EDTA/PBA disks suggested KPC or KPC plus MBL production. (**D**) No growth towards both disks indicated MßL carbapenemase production or carbapenem non-susceptibility due to ESBL/AmpC production plus porin loss.

A 0.1 M ethylenediaminetetraacetic acid (EDTA) stock solution was prepared by dissolving anhydrous EDTA in distilled water [30]. From this, 10 µL (292 µg of EDTA) was dispensed onto two sets of blank paper disks. A PBA stock solution was made by dissolving phenyl boronic acid (PBA) in dimethyl sulfoxide and water at 60 mg/mL [31]. From this, 10 µL (600 µg of PBA) was added to one set of EDTA disks. The disks were dried and used within 60 min. First, a 10 µg imipenem disk was placed on a Mueller–Hinton agar plate with lawn culture of carbapenem-susceptible *E. coli* ATCC 25922 (0.5 McFarland turbidity). Four to six colonies of the test microorganism were applied to the EDTA and EDTA plus PBA disks, which were then placed inoculum-side down adjacent to the imipenem disk. The plate was incubated overnight at 35 °C in ambient air. After 18 h, the plates were examined for an indentation or flattening of the inhibition zone. Indentation towards both EDTA and EDTA/PBA disks indicated OXA-48 carbapenemase production. Indentation towards the EDTA disk but not the EDTA/PBA disks suggested KPC or KPC plus MBL production. No growth towards both disks indicated MßL carbapenemase production or carbapenem non-susceptibility due to ESBL/AmpC production plus porin loss [14] (Figure 3).

#### 2.6.2. MASTDISCS^®^ Combi (D72C): AmpC, ESBL, and Carbapenemase Detection Set

The AmpC, ESBL, and Carbapenemase Detection Set D72C (Mast Group Ltd., UK) detects ESBL, AmpC (both chromosomal- and plasmid-mediated), and the presence of CPE in Enterobacterales without specifying the CPE type. This set includes six cartridges: cartridge A (cefpodoxime 10 μg), cartridge B (cefpodoxime 10 μg + ESBL inhibitor), cartridge C (cefpodoxime 10 μg + AmpC inhibitor), cartridge D (cefpodoxime 10 μg + ESBL inhibitor + AmpC inhibitor), cartridge E (cefpodoxime 10 μg + ESBL inhibitor + AmpC inducer), and cartridge F (penem antibiotic). The procedure was performed, and the results were analyzed in accordance with the manufacturer’s instructions [32].

#### 2.6.3. MASTDISCS^®^ Combi Carba Plus (D73C)

The Carba plus (D73C) (Mast Group Ltd., UK) detects the CPE type in Enterobacterales. This set includes five cartridges: cartridge A (penem), cartridge B (penem + MßL inhibitor), cartridge C (penem + KPC inhibitor), cartridge D (penem + AmpC inhibitor), and cartridge E (Temocillin + MßL inhibitor). The procedure was executed, and the results were analyzed in accordance with the manufacturer’s instructions.

#### 2.6.4. Immunochromatographic Assay (ICT)

**Figure 4:**
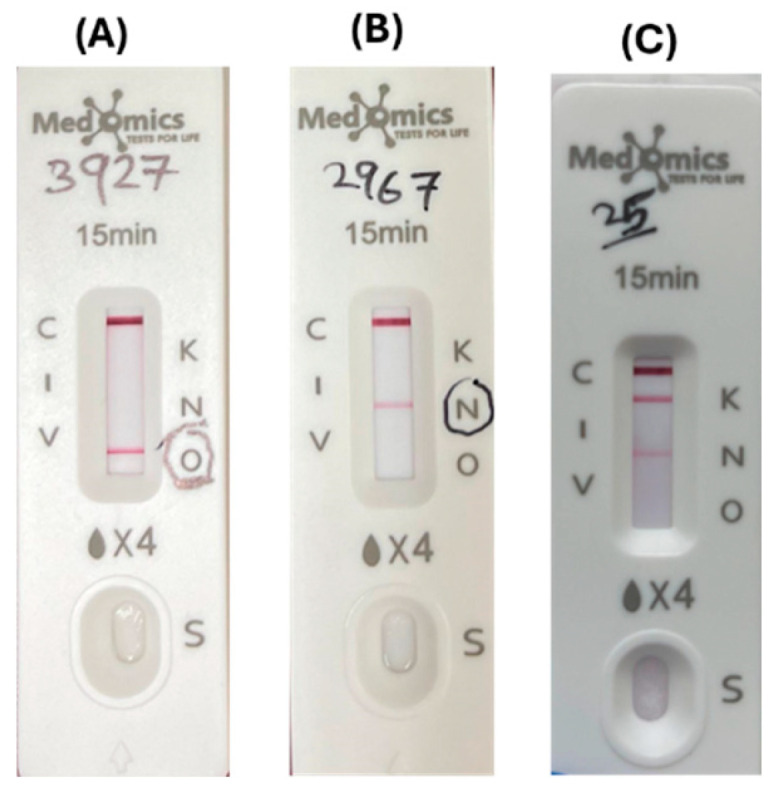
Immunochromatographic assay (ICT): (**A**) O line indicated positive result for OXA-48 carbapenemase; (**B**) N line indicated positive result for NDM carbapenemase; and (**C**) K line and N line indicated positive results for both KPC + NDM.

The KPC/IMP/NDM/VIM/OXA-48 Combo test kit (Medomics, Nanjing, China) is a lateral flow colloidal gold immunochromatographic assay (LFIA) designed to specifically detect the five most common carbapenemases (KPC, IMP, NDM, VIM, and OXA-48) using a double-antibody sandwich method [33,34,35]. The procedure was carried out and the results were interpreted according to the manufacturer’s guidelines (Figure 4).

### 2.7. Statistical Analysis

Data analysis was carried out by IBM SPSS Statistics for Windows Version 25.0. (IBM Corp., 2017, Chicago, IL, USA). A chi-square test was used to compare categorical data between two groups. Cross-tabulation was used, taking WGS, Xpert ^®^Carba-R, and PCR as gold standards to determine the sensitivity and specificity. Positive predictive values (PPVs) and negative predictive values (NPVs) were calculated using the following formulae: PPV = [True Positives (TPs)/[True Positives (TPs) + False Positives (FPs)]] × 100, NPV = [True Negatives (TNs)/[True Negatives (TNs) + False Negatives (FNs)]] × 100. PPV represents the proportion of positive test results that are true positives, while NPV represents the proportion of negative test results that are true negatives [36].

## 3. Results

The screening and confirmatory tests were evaluated against Xpert ^®^Carba-R/PCR and WGS confirmed carbapenemase (OXA-48-, KPC-, and NDM-like enzymes)-producing strains.

### 3.1. Screening Tests

D71C identified all except one isolate as a carbapenemase producer. The temocillin disk test identified OXA-48 in all 40 (100%) OXA-48-positive strains. KPC and NDM were identified in 18/20 (90%) strains each. (Table 1) All OXA-232 were also identified as OXA-48, while 4/6 isolates co-harboring KPC-2 and NDM-1 were identified as KPC. Only one out of six NDM-5 isolates was identified as NDM (Table 2). OXA-48-like producers demonstrated complete temocillin resistance (inhibition zone: 6 mm), far below the cutoff of 11 mm. It was observed that the KPCs and NDMs also demonstrated inhibition zones below the cutoff values, but near 11 mm [17].

### 3.2. Confirmatory Tests

#### 3.2.1. OXA-48 Disk Test

This test correctly identified 38/40 (95%) isolates as OXA-48-like, exhibiting high sensitivity (100% and 92.5%) and specificity (91.67% and 100%) with WGS and Xpert ^®^Carba-R/PCR as gold standards, respectively, as seen in Table 3. The negative predictive value (NPV) was 100% for WGS and 93.02% for Xpert ^®^Carba-R/PCR. Two false-positive isolates producing OXA-48 that tested negative for OXA-232 were identified as OXA-48 in 22/25 (88%) cases. It correctly identified all NDMs and KPCs. Five of the six isolates co-harboring NDM-1 and KPC-2 were identified as KPC, and all NDM-5s were identified as NDM. Overall, the OXA-48 disk test showed high sensitivity across all groups: OXA-48: 38/40 (95%); KPC and NDM: 20/20 (100%).

#### 3.2.2. D72C Test

The D72C test detected the presence of a carbapenemase in 79/80 (OXA-48-, KPC-, and NDM-like) isolates as well as 25 OXA-232, 6 NDM-5, and 6 isolates co-carrying NDM-1 and KPC-2, missing only one OXA-48-like *K. pneumoniae*. It had high sensitivity (100% with WGS as the gold standard and 97.50% with Xpert ^®^Carba-R/PCR). Specificity and PPV were 100% for both, while NPV was 100% and 97.5% for both.

#### 3.2.3. D73C Test

D73C showed slightly higher sensitivity (95%) compared to the OXA-48 disk test (92.5%) with Xpert ^®^Carba-R/PCR but lower (96%) with WGS. In comparison to the OXA-48 disk test, D73C had lower specificity (91.67%) across both gold standards. PPV was 96.15% and 95%, and NPV was 91.67% for both gold standards. Similar to the OXA-48 disk test, this test missed one OXA-232 and one isolate, which co-harbored NDM-1 and KPC-2.

#### 3.2.4. ICT Test

ICT detected all OXA-48-like isolates but missed one KPC and one NDM each. It demonstrated 100% sensitivity and NPV against both gold standards and showed 92% and 95% specificity with 96.15% and 95.24% for PPV. Among the sequenced strains, it missed one isolate that co-harbored NDM-1 and KPC-2 but identified all OXA-232 and NDM-5.

## 4. Discussion

The alarming increase in CPE is a major public health concern worldwide. For the judicious use of reserve drugs and promoting antimicrobial stewardship, timely detection of carbapenemases is essential. Balkhair et al. reported an overall prevalence rate of 10.8 (95% CI: 9.3–12.4) of multidrug-resistant Gram-negative bacteria cases per 1000 admissions [37]. The large-scale use of carbapenems in the management of extended-spectrum beta-lactamase-producing Enterobacterales has cascaded into the emergence of CPE [2,38]. The relative increase in CPE compared to the other resistance markers can have catastrophic implications for healthcare. Cloacae carried *bla*NDM, *bla*OXA48-like, and, to a lesser extent, *bla*VIM carbapenemases [39]. Initially reported in Turkey, OXA-48 prevalence has spread globally, with increasing reports in Europe, Asia, and North America [40,41]. In Oman, CPE was reported for the first time in 2011 among *Klebsiella pneumoniae* harboring NDM-1 and OXA-181 carbapenemases [42]. Other reports have described the molecular epidemiology of CPE in the country, including *Klebsiella pneumoniae*, *Escherichia coli*, and *Enterobacter.* Recent studies have reported the emergence of *bla*OXA-48, *bla*OXA-181, *bla*OXA-232, *bla*NDM-1, and *bla*NDM-5 genes [43,44,45,46]. The KPC-producing Enterobacterales were rarely reported in the GCC and Eastern Mediterranean Region, with minor incidents being reported from Saudi Arabia, Kuwait, and Qatar. In Oman, KPC producing *K. pneumoniae* were first reported in 2019. Co-carriage of *bla*KPC-2 and NDMs emerged soon after [47] (unpublished data).

Thus, identifying the precise carbapenemase enzyme is essential for optimizing patient care and promoting appropriate antimicrobial stewardship efforts. In many parts of the world, and particularly the Middle East, OXA-48-like carbapenemases predominate, followed by NDM and KPC [41]. Amongst them, the phenotypic detection of OXA-48-like carbapenemase is particularly challenging. The low-level carbapenem resistance makes it especially difficult to identify, and the diagnostic microbiology laboratories usually do not detect OXA-48-like enzymes. This leads to not only an underestimation of the incidence of OXA-48-like-producing CPE, but it also leads to inappropriate treatment. While Xpert ^®^Carba-R/PCR and WGS are standard tools for detecting them, they are expensive and not readily available in most diagnostic laboratories, particularly in the low- and medium-income countries (LMICs). In this study, we tested commercial and in-house screening and confirmatory tests of varying costs and assessed their utility in detecting different carbapenemases with a particular focus on OXA-48-like genes.

The temocillin disk test proved to be a good, simple, and cheap screening test for carbapenemases, with a good discriminatory index in detecting OXA-48. It exhibited 100% sensitivity for OXA-48 detection and 90% for KPCs and NDMs [28]. The expression of high-level temocillin resistance along with a lack of inhibition by boronic acid, dipicolinic acid, and EDTA is a cheap and effective way to identify OXA-48-like carbapenemases [48]. On the other hand, D71C achieved 100% sensitivity for the latter two and 97.5% for OXA-48-like carbapenemases [35].

This study reveals that the OXA-48 disk test is a reliable and cost-effective method for detecting carbapenemases, in general, and OXA-48 in particular. With high sensitivity (100% and 92.5%) and specificity (91.67% and 100%) when compared to two gold standards—WGS and Xpert ^®^Carba-R/PCR—the OXA-48 disk test is a valuable tool for detecting OXA-48, KPC, and NDM carbapenemases. This test can be easily validated and implemented in clinical laboratories. Others have reported this test to be an excellent tool in resource-constrained settings for promoting antimicrobial stewardship [31]. Tsakris et al. reported a 96.3% sensitivity and 97.7% specificity [31]. However, Koroska et al. reported a lower sensitivity of 53.6%, which improved to 98.8% when a high inoculum was used [35]. In this study, too, a higher inoculum (4–6 colonies) yielded better results. However, the OXA-48 disk test needs to be validated in individual laboratories, which may be time-consuming, and correct interpretations would require experience. This test is based on the utilization of EDTA to permeabilize bacterial cells, releasing β-lactamases into the environment. EDTA also inhibits MBL carbapenemases, while PBA inhibits KPCs. A poor detection rate of the co-carriage of dual carbapenemases is another limitation of this test [35].

While the D72C test is an excellent tool for detecting ESBL, AmpC, and carbapenemases with good reproducibility and discrimination, it does not identify the type of carbapenemase, which is a disadvantage. For detecting carbapenemases in general, it had high sensitivity (100% and 97.50%) with WGS and GeneXpert gold standards, with 100% specificity. Another study reported a lower sensitivity (90%) for carbapenemase detection [14]. In a comparative evaluation of various phenotypic tests, the D72C test achieved an accuracy of 95.4% for detecting AmpC and carbapenemase production [15].

Multi-disk diffusion tests, based on the synergy between β-lactamase inhibitors and carbapenems, effectively distinguish between various carbapenemases. The inhibitor for the detection of KPC is boronic acid, and dipicolinic acid or ethylenediaminetetraacetic acid (EDTA) is used for MBL detection. Clavulanate (an inhibitor of ESBL), cloxacillin (an inhibitor of AmpC), and avibactam (NXL104) are useful for OXA-48 detection. The D73C test supersedes D71C entirely, and if used in conjunction with D72C, which detects ESBL and AmpC as well, it could promote antimicrobial stewardship considerably. Although the combination of these two tests (D71C and D72C) does increase the cost, the benefit of identifying ESBL and AmpC and instituting appropriate antimicrobials may outweigh the incurred cost. However, ESBL and AmpC can be detected by cheap, excellent, and easy in-house tests as well. Al Mamari et al. demonstrated that D73C was slightly inferior to the OXA-48 disk test with 96% sensitivity and 100% specificity with WGS as the gold standard [15]. However, it has an advantage over D71C and D72C in that it identifies the carbapenemase types. Al-Zahrani et al. (2018) [49] reported that this method demonstrated a good discriminatory index in identifying KPC and MBL among *K. pneumoniae*, but it was not satisfactory in distinguishing between OXA-48-type genes and different MBL genes such as NDM, VIM, and IMP. All (100%) OXA-48 and OXA-232 were identified as OXA-48-like. They demonstrated high sensitivity (97.5% for OXA-48 and 100% for NDM) and 91.67% specificity, making them highly accurate. The negative predictive values were comparable, although they were lower in D73C (91.67%), indicating that while both tests are effective, the OXA-48 disk test may offer more reliable results overall, once appropriately standardized.

ICT, with 100% sensitivity and 95% specificity, surpassed both the in-house OXA-48 disk test and the D73C in performance, making it the gold standard among phenotypic tests. Compared to Xpert ^®^Carba-R, ICT is superior in specificity but slightly weaker in sensitivity. With other commercial phenotypic tests being time-consuming, ICT provides rapid results within 20 min at a moderate cost. However, it must be borne in mind that the time to detection of CPE will be a minimum of 24 h and may extend to 48 h if the isolate is not pure for ICT and GeneXpert, while it will take a minimum of 48 h for the other phenotypic tests. Molecular tests with comparable sensitivities and specificities are expensive and not widely available in most parts of the world [17]. Several good ICTs are available in the market, like the NG-Test^®^ DetecTooL (CARBA-5, NG Biotech, Guipry-Messac, France, KINVO (Medomics Medical Technology, Nanjing, Jiangsu, China), the O.K.N.V.I RESIST-5 (RESIST-5, Coris BioConcept, Gembloux, Belgium), and the KPC and OXA-48 K-set. ICTs demonstrated high performance with excellent accuracy for the rapid identification of KPC and OXA-48 enzymes [16,50]. Of course, for maximal detection accuracy, WGS remains the ideal gold standard, although Xpert ^®^Carba-R and ICT have excellent sensitivity, specificity, reproducibility, and discrimination [51].

## 5. Conclusions

Overall, the phenotypic tests were cheaper and demonstrated excellent sensitivity and specificity but had a longer turnaround time. Temocillin offers a degree of discrimination of carbapenemase type, especially for OXA-48, and we recommend that it may be incorporated in the laboratory as a screening tool. ICT with excellent sensitivity and specificity, rapid results, ease of performance, and relatively low cost appears to be an excellent tool for the detection of carbapenemases in diagnostic laboratories. This test format has the potential to be a game-changer in optimizing patient care and promoting antimicrobial stewardship at scale. We report on one kit here; however, there are several validated ones on the market. ICTs should be further evaluated for their post-market performance and lot-by-lot variation. The OXA-48 disk test is an excellent tool too and will serve well in resource-constrained centers. We believe that in-house screening tools and confirmatory tests are both as equally sensitive and specific as the commercial tests. Hence, depending on the resources, laboratories may exercise their discretion and adopt appropriate tests to identify the carbapenemase types and promote antimicrobial stewardship and optimum patient management.

## Figures and Tables

**Table 1:** Assessment of phenotypic tests for detection of OXA-48, KPC and NDM in *Klebsiella pneumoniae* using Xpert ®Carba-R/PCR as gold standard.

Carbapenemase	Gold Standard	Screening Test		Confirmatory Tests			
				*In-House*	*Commercial Tests*		
	Xpert Carba-R/PCR	Temocillin Disc Test	D71C	OXA-48 Disk Test	D72C	D73C (*n* = 32)	ICT
	*N* (%)	*N* (%)	*N* (%)	*N* (%)	*N* (%)	*N* (%)	*N* (%)
**OXA-48 (*n* = 40)**	40 (100%)	40 (100%)	39 (97.5%)	38 (95%)	39 (97.5%)	19 (95%) *	40 (97.5%)
**KPCs (*n* = 20)**	20 (100%)	18 (90%)	20 (100%)	20 (100%)	20 (100%)	5 (83.3%) **	19 (95%)
**NDM (*n* = 20)**	20 (100%)	18 (90%)	20 (100%)	20 (100%)	20 (100%)	6 (100%) ***	19 (95%)

* Total number of OXA-48 tested by D73 C were 20 isolates. ** Total number of KPCs tested by D73C were 6 isolates. *** Total number of NDMs tested by D73C were 6 isolates.

**Table 2:** Assessment of phenotypic tests for detection of OXA-232, KPC-2+NDM-1 and NDM-5 in *Klebsiella pneumoniae* using WGS as gold standard.

Carbapenemase	Gold Standard	Screening Test		Confirmatory Tests					Extended Spectrum β-Lactam Genes
				*In-House*	*Commercial Tests*				
	WGS	Temocillin Disk Test	D71C	OXA-48 Disk Test	D72C	D73C (*n* = 32)	ICT	Xpert Carba-R/PCR	
	*N* (%)	*N* (%)	*N* (%)	*N* (%)	*N* (%)	*N* (%)	*N* (%)	*N* (%)	
**OXA-232 (*n* = 25)**	25 (100%)	25 (100%)	25 (100%)	22 (88%)	25 (100%)	19 (95%) *	20 (100%)	25 (100%)	CTX-M-15, SHV-11, SHV-28, SHV-67, SHV-75. SHV106, SHV-182
**KPC-2+NDM-1 (*n* = 6)**	6 (100%)	2 (33.3%)	6 (100%)	5 (83.3%)	6 (100%)	5 (83.3%) **	5 (83.3%)	6 (100%)	CTMX-65, SHV-12, SHV-12 and SHV-182 and SHV-182
**NDM-5 (*n* = 6)**	6 (100%)	1 (20%)	6 (100%)	6 (100%)	6 (100%)	6 (100%) ***	6 (100%)	6 (100%)	CTX-M-65, TEM-1B, TEM1C, SHV-11, SHV-12, SHV-67, SHV-75

* Total number of OXA-48 tested by D73 C were 20 isolates. ** Total number of KPCs tested by D73C were 6 isolates. *** Total number of NDMs tested by D73C were 6 isolates.

**Table 3:** Sensitivity and specificity of phenotypic tests for detection of OXA-48 like, KPC and NDM using WGS, Xpert ®Carba-R and ICT as the gold standards.

Gold Standards		Screening Test (%)		Confirmatory Tests (%)			
				*In-House Test*	*Commercial Tests*		
		Temocillin	D71C	OXA-48 Disk	D72C	D73C	ICT
**WGS (*n* = 37)**	**SN (%)**	100	100	100	100	96.15	100
	**SP (%)**	76.92	100	91.67	100	91.67	92.31
	**PPV (%)**	89.29	100	95.65	100	96.15	96.15
	**NPV (%)**	100	100	100	100	91.67	100
**X-pert Carba-R/PCR (*n* = 80)**	**SN (%)**	100	97.5	92.5	97.5	95	100
	**SP (%)**	70	100	100	100	91.67	95
	**PPV (%)**	76.92	100	100	100	95	95.24
	**NPV (%)**	100	97.56	93.02	97.56	91.67	100

SN = Sensitivity; SP = Specificity; PPV = Positive predictive value; NPV = Negative predictive value.

## Data Availability

The original contributions presented in this study are included in the article/Supplementary Materials. Further inquiries can be directed to the corresponding author.

## Supplementary Materials

The following supporting information can be downloaded at https://www.mdpi.com/article/10.3390/diagnostics15080949/s1: Table S1. Preparation of Multiplex PCR, Table S2. Positive Controls Used for the CRE Genes, Table S3. PCR Conditions, Table S4. Comparison of cost of tests per isolate.
