# Supplementary material for: Assessment of Phenotypic Tools for Detection of OXA-48, KPC, and NDM in Klebsiella pneumoniae in Oman
Source: Diagnostics (Basel). 2025 Apr 8;15(8):949. doi: 10.3390/diagnostics15080949 (PMC12025575; doi:10.3390/diagnostics15080949)
Supplement: Supplementary file 1 [file diagnostics-15-00949-s001.zip › Supplementary Table S1.pdf]

| Supplementary Table S1: Preparation of Multiplex PCR | |
|---|---|
| | **Multiplex PCR** |
| **Reagent** | **Volume ( µl )/ Sample (1X)** |
| N-$H_2O$ | 2.5 |
| Platinum Master Mix | 12.5 |
| NDM-R | 1 |
| NDM-F | 1 |
| OXA-R | 1.25 |
| OXA-F | 1.25 |
| KPC-R | 0.75 |
| KPC-F | 0.75 |
| VIM-R | 0.75 |
| VIM-F | 0.75 |
| IMP-R | 0.75 |
| IMP-F | 0.75 |
| **Total volume** | 24 |
| **DNA extract** | **1** |
| **Final volume** | **25** |
