# Supplementary material for: Assessment of Phenotypic Tools for Detection of OXA-48, KPC, and NDM in Klebsiella pneumoniae in Oman
Source: Diagnostics (Basel). 2025 Apr 8;15(8):949. doi: 10.3390/diagnostics15080949 (PMC12025575; doi:10.3390/diagnostics15080949)
Supplement: Supplementary file 1 [file diagnostics-15-00949-s001.zip › Supplementary Table S2.pdf]

Supplementary Table S2: Positive Controls Used for the CRE Genes

Gene	NDM	VIM	IMP	KPC	OXA-48
**Positive control**	*Klebsiella pneumonia*	*Pseudomonas aeruginosa*	*Pseudomonas aeruginosa*	*Klebsiella pneumonia*	*Klebsiella pneumonia*
**Band size (bp)**	782	389	587	900	438
