# Supplementary material for: Assessment of Phenotypic Tools for Detection of OXA-48, KPC, and NDM in Klebsiella pneumoniae in Oman
Source: Diagnostics (Basel). 2025 Apr 8;15(8):949. doi: 10.3390/diagnostics15080949 (PMC12025575; doi:10.3390/diagnostics15080949)
Supplement: Supplementary file 1 [file diagnostics-15-00949-s001.zip › Supplementary Table S3.pdf]

Supplementary Table S3: PCR Conditions

**PCR Condition**			
**Steps**	**No. Cycle**	**Temperature (° C)**	**Time**
**Denaturation**	1	95	5min
		95	45sec
**Annealing**	35	60	45sec
		72	1min
**Elongation**	1	72	8min
