# Supplementary material for: Assessment of Phenotypic Tools for Detection of OXA-48, KPC, and NDM in Klebsiella pneumoniae in Oman
Source: Diagnostics (Basel). 2025 Apr 8;15(8):949. doi: 10.3390/diagnostics15080949 (PMC12025575; doi:10.3390/diagnostics15080949)
Supplement: Supplementary file 1 [file diagnostics-15-00949-s001.zip › Supplementary Table S4.pdf]

Supplementary Table S4. Comparison of cost of tests per isolate

**Test**	**Cost**	
	(OMR)	($)
D71C	0.756	1.96
D73C	0.942	2.45
D72C	1.176	3.05
ICT	5	12.99
PCR	15	38.96
Xpert Carba-R	26.5	68.83
WGS	50.55	105.32
